# Medicinal Leech CNS as a Model for Exosome Studies in the Crosstalk between Microglia and Neurons

**DOI:** 10.3390/ijms19124124

**Published:** 2018-12-19

**Authors:** Antonella Raffo-Romero, Tanina Arab, Issa S. Al-Amri, Francoise Le Marrec-Croq, Christelle Van Camp, Quentin Lemaire, Michel Salzet, Jacopo Vizioli, Pierre-Eric Sautiere, Christophe Lefebvre

**Affiliations:** 1 U1192-Laboratoire Protéomique, Réponse Inflammatoire et Spectrométrie de Masse (PRISM), Univ. Lille, INSERM, F-59000 Lille, France; anto_aqp@hotmail.com (A.R.-R.); tanina.arab@univ-lille.fr (T.A.); francoise.croq@univ-lille.fr (F.L.M.-C.); christelle.van-camp@univ-lille.fr (C.V.C.); quentin.lemaire@univ-lille.fr (Q.L.); michel.salzet@univ-lille.fr (M.S.); jacopo.vizioli@univ-lille.fr (J.V.); 2 DARIS Centre for Scientific Research and Technology Development, University of Nizwa, P.O. Box 33, Birkat Al-Mouz, PC 616 Nizwa, Oman; issa.alamri@unizwa.edu.om

**Keywords:** microglia, exosomes, neurite outgrowth, leech

## Abstract

In healthy or pathological brains, the neuroinflammatory state is supported by a strong communication involving microglia and neurons. Recent studies indicate that extracellular vesicles (EVs), including exosomes and microvesicles, play a key role in the physiological interactions between cells allowing central nervous system (CNS) development and/or integrity. The present report used medicinal leech CNS to investigate microglia/neuron crosstalk from ex vivo approaches as well as primary cultures. The results demonstrated a large production of exosomes from microglia. Their incubation to primary neuronal cultures showed a strong interaction with neurites. In addition, neurite outgrowth assays demonstrated microglia exosomes to exhibit significant neurotrophic activities using at least a Transforming Growth Factor beta (TGF-β) family member, called nGDF (nervous Growth/Differentiation Factor). Of interest, the results also showed an EV-mediated dialog between leech microglia and rat cells highlighting this communication to be more a matter of molecules than of species. Taken together, the present report brings a new insight into the microglia/neuron crosstalk in CNS and would help deciphering the molecular evolution of such a cell communication in brain.

## 1. Introduction

Brain disorders have a growing impact on human disability and morbidity worldwide. Although the etiology of most brain diseases is poorly described, it is clear that pro-inflammatory events in the central nervous system (CNS) lead to neurodegenerative mechanisms and cognitive decline. The neuroinflammatory state in the CNS is a broad process involving microglia cells as resident immune cells [1]. Blood immune cells can be recruited in turn into the brain as a consequence of the blood brain barrier disrupture. But the interactions between microglia subpopulations and neurons basically represent a key process of CNS integrity and neuroinflammatory regulations [2,3,4,5].

The medicinal leech (*Hirudo medicinalis*) CNS is an interesting model in this regard because microglia cells can be studied in close relation with either neuronal cell bodies or axons. Indeed, neuronal cell bodies are concentrated in ganglia and project the axons into the connective tissues [6,7]. Microglia subpopulations are located in ganglia as well as connective tissues so that they can distinctly interact with neuronal cell bodies or axons, respectively. Thus, it is possible to only injure axons in the middle of connectives to study the responses of the neuronal cell bodies leading to the axonal sprouting and synaptogenesis. As their mammalian counterparts, microglia cells in leech can be followed using iba1 as a specific marker [8]. They change their morphology from ramified to amoeboid shape upon activation, thus facilitating their recruitment towards lesioned areas [7]. Microglia are the only migrating cells to be recruited at the injury site within 24 h post-lesion [9,10] and promote a regenerative process [11,12]. Indeed, if microglia accumulation is delayed at lesions, the axonal sprouting is consequently affected [13]. It shows the importance of a neuroprotective crosstalk between damaged neurons and activated microglia in connectives. Numerous chemotactic signals including adenosine triphosphate (ATP), complement factor C1q, endothelial-monocyte-activating polypeptide II (EMAPII), and interleukin-16 (IL-16) play a role in this microglia accumulation [14,15,16,17,18,19]. However, the specific functions of recruited microglial cells throughout the response to injury are still to be elucidated. Otherwise, in response to injury, other microglial cells naturally located in ganglia simultaneously dialog with neuronal cell bodies [8].

Consequently, elucidating the processes contributing to the crosstalk between neurons and microglia will help to understand the chronological events following a lesion and would give a new insight into the refinement of microglia-neuron interactions. In this context, the present report describes the production of extracellular vesicles (EVs) from microglia and their potential role in neuron–microglia crosstalk.

Extracellular vesicles are lipid bilayered membrane vesicles, presenting a diameter from 30 to 1000 nm, involved in cell-to-cell communication. Two EV populations are currently described: exosomes and microvesicles [20]. Exosomes are 30–100 nm in diameter and are synthesized within the endosomal system and then secreted by fusion of multivesicular bodies with plasma membrane. Microvesicles, also known as ectosomes, are 50–1000 nm in diameter and result from a budding process of plasma membrane [21]. Extracellular vesicles are considered as molecular cargos including proteins, lipids, and nucleic acids [22,23,24]. Because exosomes and microvesicles have an overlapping size and present molecular similarities, they remain difficult to be structurally and biologically discriminated [25]. There is at present a growing interest either in physiological or in pathological ways to better understand their ability to affect recipient cells, especially in CNS functions [26]. Because they reflect the functional profile of producing cells, EVs can be used as biomarkers for pathologies [27,28]. Their modification would also lead to create original therapeutic cargos able to cross the blood brain barrier [27,29].

Recent studies indicate that EVs play a key role in the physiological interactions between glia cells and neurons leading to a correct CNS development and/or integrity [26,30,31]. Of interest, microglia have also been described to generate and release EVs in physiological and pathological conditions [32,33]. In this context, understanding the functions of EVs from microglia may contribute to specify the role of microglia subpopulations in the control of neuroinflammation. The present study shows the production and release of EVs from microglia in the leech CNS. Those EVs closely interact with neurons and exhibit neurotrophic properties in vitro, especially using a member of the TGF-β family. Taken together, the results also show the evolutionary conservation of EV-mediated dialog between leech microglia and rat recipient cells.

## 2. Results

**Figure 1:**
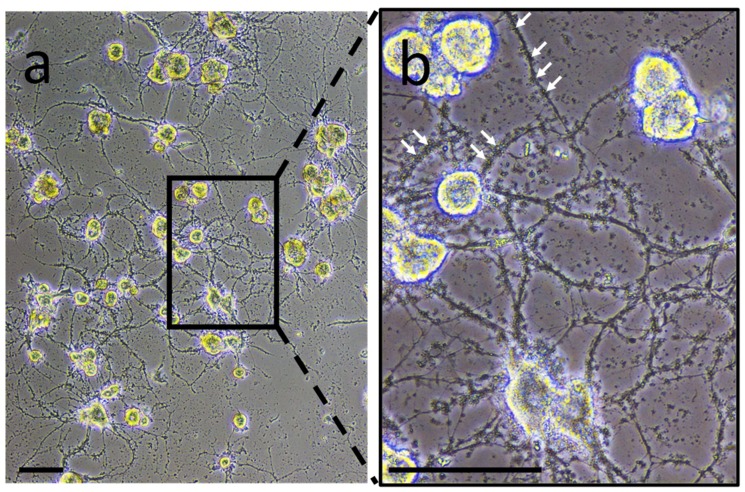
Neurons and microglia primary co-culture. (**a**) During the co-culture, adherent neurons exhibit neurite outgrowth while activated microglial cells are still floating. The renewal of the culture medium washed away the microglial cells while maintaining the neurons and some products released from both cell populations. (**b**) Enlargement showing vesicle-like structures (white arrows as examples) interacting with neurites. Scale bars correspond to 50 µm.

The structural features of the nerve chain in *H. medicinalis* facilitate interaction studies between microglia and neurons by taking into account that there are no comparable glial cells such as astrocytes or oligodendrocytes [6]. Moreover, since this annelid does not regulate its body temperature, freshly collected and dissociated cells show a high resistance in vitro and can be maintained in primary culture at room temperature and without the use of CO_2_. Experiments to characterize neuron- or microglia-associated secretory products were carried out in primary culture to collect conditioned medium. In neuron-microglia co-culture, very small structures were observed. They were comparable in size to vesicle-like structures and were closely in association to neurites developed by neurons (Figure 1). This observation suggests the possibility that such vesicles are produced and released by nerve cells. This preliminary result incited to perform the following experiments in order to confirm this hypothesis.

**Figure 2:**
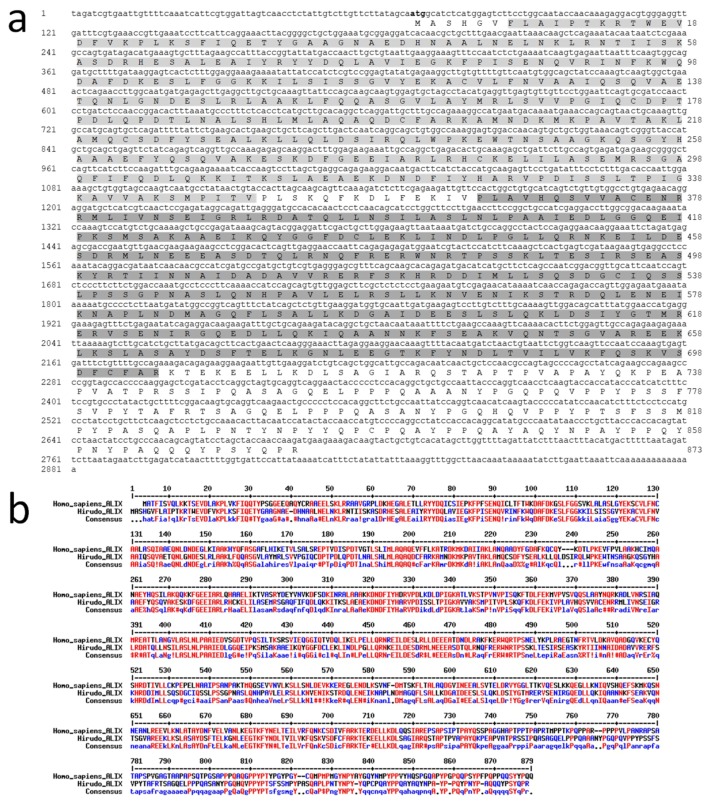
Molecular characterization of *Hirudo medicinalis* (*Hm*) Alix. (**a**) *Hm*Alix is a protein of 873 amino acids (~97 kDa) from a mRNA of 2281 nucleotides. *Hm*Alix protein is composed of N-terminal Bro1 (IPR038499, light grey) and Alix V-shaped (IPR025304, dark grey) domains, as observed in counterparts from other organisms. (**b**) The sequence alignment between *Hm*Alix and *Homo sapiens* Alix forms shows high and low consensus homologies (red and blue residues, respectively) which allows using polyclonal anti-human Alix antibodies to detect the protein in the leech central nervous system (CNS).

These observations and recent studies showing the production of EVs in the CNS motivated to look at such structures in the leech nerve chain by using antibodies directed against EV-specific molecules. Among those molecules, the analysis of leech databases allowed identifying a sequence coding for a *Hirudo medicinalis* (*Hm*) form of the EV marker Alix [34], a cytoplasmic protein previously demonstrated to be highly present in exosomes [35]. *Hm*Alix is a protein of 873 amino acids (~97 kDa) from a mRNA of 2281 nucleotides (Figure 2a). The protein is composed of N-terminal Bro1 (IPR038499) and Alix V-shaped (IPR025304) domains in accordance to Alix molecules from other organisms [36]. *Hm*Alix exhibits an amino acid sequence 49% identical and 68% homologous to its human counterpart (Figure 2b). Similar homologies were observed to Alix sequences from other animal organisms.

**Figure 3:**
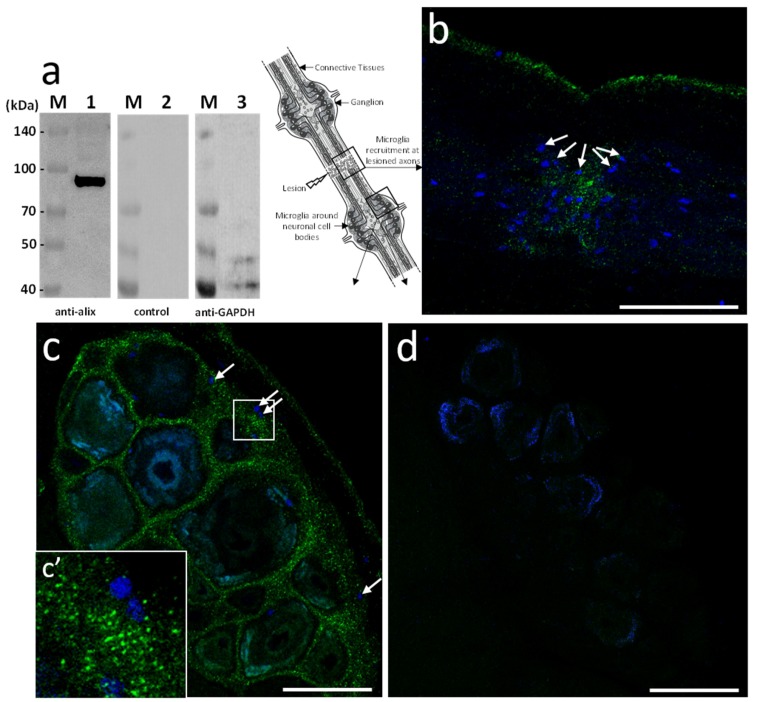
*Hm*Alix immunodetection in the leech CNS. (**a**) Western blotting analyses from leech CNS protein extracts using mouse polyclonal anti-human Alix antibodies reveals a 97 kDa protein (lane 1) compared to secondary antibody alone (lane 2) as a negative control. This membrane was stripped and incubated with anti-Glyceraldehyde 3-phosphate dehydrogenase (GAPDH) antibody as a loading control (lane 3). Molecular weights of ladder lanes (M) are reported. (**b**–**d**) From nerve cord fragment (see diagram), confocal microscopy analyses using mouse polyclonal anti-human Alix antibodies were performed 24 h following a connective crush. (**b**) In connectives, the immunofluorescence (green) showed a signal at lesioned axons where microglia may be recruited as shown using nuclear counterstaining with Hoechst 33342 (blue) (see arrows as examples). (**c**,**c’**) In ganglia, the immunofluorescence (green) showed a signal in interneuronal spaces (see 3.7-fold magnification in c’). A few microglia nuclei are visible at this focal plane (arrows). (**d**) No signal was detected in CNS treated only with secondary antibody as negative control. Cell nuclei were counterstained in blue. Scale bars correspond to 50 µm.

Based on the sequence homology, mouse polyclonal anti-human Alix antibodies were used in further studies. The immunoblot results showed the detection of a unique 97 kDa product in the leech CNS corresponding to the expected size of the predicted protein (Figure 3a). Then, ex vivo studies were made in isolated fragments of CNS (see diagram) after an axonal lesion and allowed locating Alix-positive vesicles. Indeed, the results showed Alix-positive nanostructures at lesions (Figure 3b) as well as in ganglia between neuronal cell bodies (Figure 3c). No signal was observed using secondary antibody alone as negative control (Figure 3d) confirming the specificity of the immunodetections (Figure 3a–c). Thus, the results corroborate the hypothesis previously emitted (Figure 1) suggesting that some nanostructures released by neurons or microglia are indeed EVs. Of interest, the immunopositive signal for Alix in connectives was collocated to the microglia recruitment at the injury site (Figure 3b) and was also observed in interneuronal spaces, the natural location of ganglionic microglia (Figure 3c,c’).

**Figure 4:**
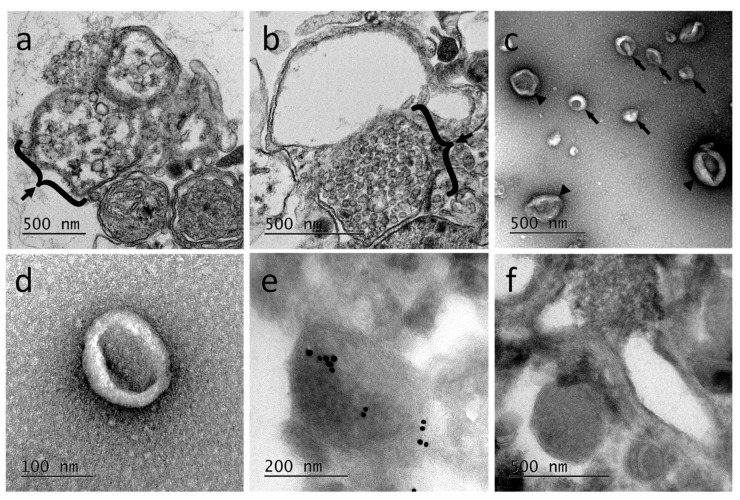
Transmission electron microcopy (TEM) of leech microglial EVs. (**a**,**b**) Cryosections of isolated microglia containing into the cytoplasm multivesicular bodies (braces), structures typical of exosome biogenesis. (**c**,**d**) Morphological analyses of microglia-released extracellular vesicles collected by differential centrifugation of primary microglia conditioned medium. These EV-enriched fractions revealed the presence of vesicles from 50 nm (arrows) to 300 nm (arrowheads) in diameter (**c**), most of which are around 100 nm (**d**). Immuno-gold labeling performed on cryosections from the extracellular vesicles (EV)-enriched samples showed the presence of some EVs positive to primary anti-human Alix antibodies (**e**). Negative controls were performed incubating EV sections with secondary antibodies alone (**f**).

Ultrastructural studies using transmission electron microcopy (TEM) were performed from primary microglia cultures. The results revealed the presence of multivesicular bodies, typical of exosomal biogenesis, in those cells demonstrating at least the production of exosomes in microglia (Figure 4a,b). Extracellular vesicles released from primary microglial cells were subsequently purified. The EV-enriched fractions showed vesicle populations ranging from 50 to 300 nm (Figure 4c) with a higher population around 100 nm (Figure 4d). The EV-enriched fractions obtained by differential centrifugation were frozen and sectioned for immune-electron microscopy treatment. The observation of cryosections by TEM (Figure 4e,f) confirmed the presence in the sample of EVs having different sizes, as described for Figure 4c. The immunogold staining was performed using primary anti-human Alix antibodies. Results showed some positively labeled EVs confirming that Alix is a cargo component of leech microglial EVs. Control experiments were performed using the secondary antibodies alone (Figure 4f). Therefore, microglia are able in the leech CNS to produce and release a high number of extracellular vesicles, some of which being exosomes.

**Figure 5:**
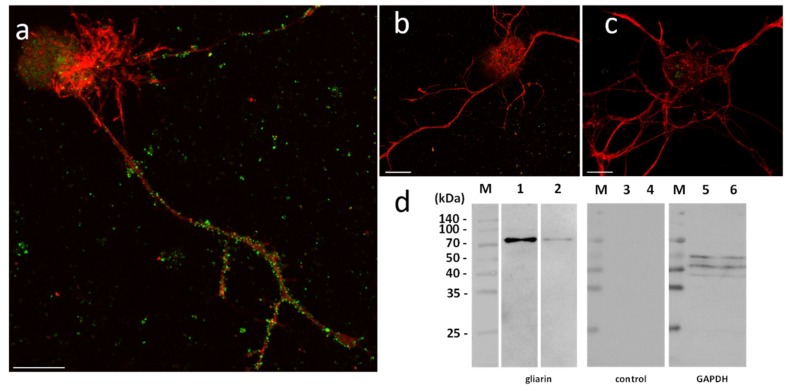
Immunofluorescence analyses of leech nerve cell cultures. (**a**) Neuron-microglia co-cultures immunostaining with mouse monoclonal anti-gliarin antibody revealed the presence of positively stained vesicles (green) associated to neurites. (**b**) The immunostaining with anti-gliarin did not reveal the presence of immunopositive vesicles in a culture of leech neurons alone. (**c**) No specific signal was observed in the same culture treated with the secondary antibody alone as control. Neuron cell bodies were counterstained with rhodamine-conjugated phalloidin (red). Scale bars correspond to 20 µm. (**d**) A unique 75 kDa positive signal was immunodetected in microglia-derived EV (lane 1) as well as in microglial cell (lane 2) protein extracts, confirming the presence of gliarin-positive EVs. No signal was observed on the same samples using secondary antibodies alone as negative control (lanes 3 and 4). Western blot analysis was performed on the same control membrane using anti-GAPDH antibody as a loading control (lanes 5 and 6). Molecular weights (M) are reported.

In order to assess their functional involvement and especially the possible use of microglial EVs in a specific dialog towards neurons, in vitro primary cultures of neurons were performed in naïve vs. microglia-activated conditions. In these experiments, due to a possible EV production by neurons, microglial EVs were not detected with Alix but using specific antibodies against gliarin, an intermediate filament protein specific in leech glial cells, a marker for microglia [8,37]. The microscopic observation of microglia-activated neurons showed a massive population of gliarin-positive EVs aggregated to neurons, especially along growing neurites (Figure 5a). No gliarin-positive nanostructure was observed in interaction with neurites in naïve neuron primary culture, demonstrating the microglial specificity and origin of those EVs (Figure 5b). Finally, the negative control only using secondary antibodies presented no EV signal in neuron cultures (Figure 5c). The controls only revealed a non-specific signal into the neuronal cell bodies (Figure 5b,c). Western blotting analyses were performed from leech microglia EV (lanes 2, 5, and 8) and leech microglia cell (lanes 3, 6, and 9) protein extracts. The use of mouse monoclonal anti-gliarin primary antibody revealed a ~75 kDa protein in both extracts (lanes 2 and 3) compared to secondary antibody alone (lanes 5 and 6) used for negative control. This membrane was then stripped to remove the immunostaining and incubated with anti-Glyceraldehyde 3-phosphate dehydrogenase (GAPDH) antibody as a loading control (lanes 8 and 9). Thus, the results show the presence of this glial marker in microglia EVs and demonstrate that microglia-specific EVs have an ability to strongly interact with neurons.

**Figure 6:**
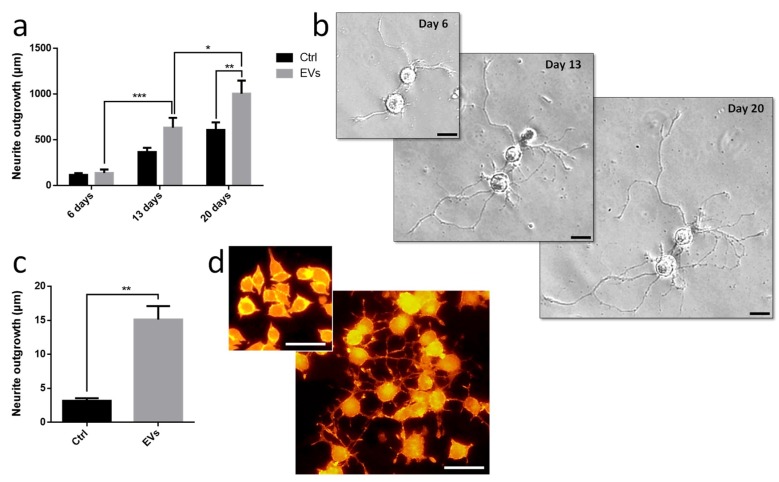
Influence of leech microglia EVs in neurite outgrowth assays. (**a**,**b**) Leech neurons were primarily cultured with either EV-enriched fractions or control supernatants from enrichment procedure. (**a**) The measures of neurite length were independently made on individual neurons at days 6, 13, and 20 showed a significant outgrowth throughout the culture under EVs and a higher outgrowth at day 20 in EV-activated condition compared to control. (**b**) The images show the same cells under EVs from day 6 to day 20. (**c**,**d**) Rat PC12 cells were cultured for only 7 days including a treatment at day 2 with either EV-enriched fractions or control supernatants. (**c**) The measures of neurite length were overall made on cell population and significantly showed a higher outgrowth under EVs compared to control. (**d**) The images show cells after 6 days treatment in control (top frame) and EV-activated (main frame) conditions. Scale bars correspond to 20 µm. Significance (* *p* < 0.05, ** *p* < 0.01, *** *p* < 0.001) was calculated by ANOVA paired *t*-test (bar represents standard errors of mean).

Leech microglia were primarily cultured to collect conditioned medium. Following the enrichment procedure, we investigated the effect of EV-enriched fraction on neurite outgrowth assays from different models. Using primary leech neurons, the results showed a significant increase of neurite outgrowth under microglial EV exposure compared to control supernatant (Figure 6a,b). Of great interest, leech microglial EVs exhibited similar effects on rat PC12 cells (Figure 6c,d). Even though these cells are not considered as adult neurons, their treatment with EVs significantly increased the production of long processes known as neurite varicosities compared to EV-free control supernatant (Figure 6c,d). These rat PC12 cells used as a model for neuronal differentiation were consequently reactive to leech microglia EVs demonstrating the evolutionary conservation of such a molecular dialog in the CNS.

**Figure 7:**
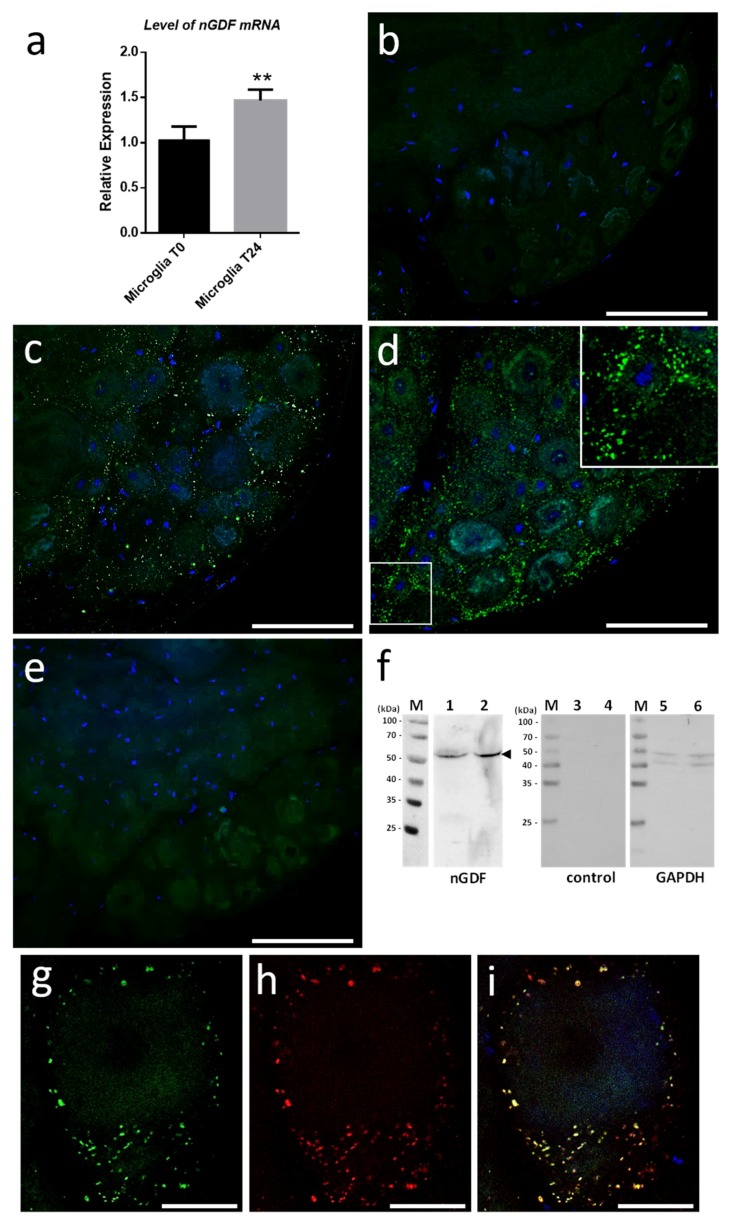
Induction of *ngdf* mRNA level and immunolocalization of nGDF protein. (**a**) q-PCR results indicate that *ngdf* mRNA is present in freshly dissociated (T0h) microglial cells and significantly increases in cultured microglia (T24h). Significance (** *p* < 0.01) was calculated by ANOVA paired *t*-test (bar represents standard errors of mean). (**b**–**e**) Confocal immunofluorescence analyses using rabbit polyclonal anti-human TGF-β1 antibodies on nerve cords freshly dissected (T0h) (**b**) or cultured ex vivo 6 h (**c**) and 24 h (**d**) following a connective crush. In ganglia, the nGDF immunostaining (green) showed a punctate signal in interneuronal spaces in accordance to the natural place of ganglionic microglia highlighted by nuclei counterstaining with Hoechst 33342 (blue). Inset in (**d**) shows a ~2-fold magnification of nGDF immunopositive vesicles surrounding neuron cell bodies. No signal was detected in nerve cord treated only with secondary antibody as negative control (**e**). Scale bars correspond to 50 µm. (**f**) Western blotting analyses from leech microglial EVs (lanes 1, 3, and 5) and cell (lanes 2, 4, and 6) protein extracts. The use of rabbit polyclonal anti-human TGF-β1 primary antibodies revealed a ~55 kDa protein in both extracts (lanes 1 and 2) compared to secondary antibody alone (lanes 3 and 4) used for negative control. This membrane was then stripped to remove the immunostaining and incubated with anti-GAPDH antibody as a loading control (lanes 5 and 6). Molecular weights of ladder lanes (M) are reported. (**g**–**i**) Double immunostaining using anti-TGF-β1 and anti-Alix antibodies on a nerve cord T24h post lesion. Several EVs surrounding a neuron cell body resulted positive for nGDF (green) (**g**) and Alix (red) (**h**). Both signals colocalize (yellow) in most of the vesicles (**i**). Cell nuclei are counterstained by Hoechst as indicated above. Scale bars correspond to 10 µm.

We started investigating the molecular contents from microglial EVs triggering neurite outgrowth. Current studies in the laboratory demonstrated the involvement of Transforming Growth Factor beta (TGF-β) signaling in the crosstalk between microglia and neurons. That is why we focused on nervous Growth/Differentiation Factor (nGDF), a leech form of TGF-β family member. The q-PCR analyses showed the increase of *ngdf* mRNA level in 24-h cultured microglia cells compared to freshly dissociated ones (T0h) (Figure 7a). To assess the presence of nGDF protein in leech CNS submitted to connective lesion and cultured ex vivo, we performed immunostaining analyses using anti-human TGF-β1 antibodies. No signal for nGDF was observed on freshly dissected nerve chains (T0h) (Figure 7b). Of interest, confocal analyses showed the immunodetection of nGDF protein in punctate nanostructures (green) in close relation to neuronal cell bodies in nerve chains cultured 6- and 24-h post lesion (Figure 7c,d). These results are in accordance to the natural position of ganglionic microglia suggesting these EV-like structures to be of microglial origin. No signal in the negative control using secondary antibody alone was observed (Figure 7e). To assess the origin of nGDF-positive vesicles, microglia EVs were enriched from isolated cells and analyzed by immunoblot using anti-TGF-β1 antibodies (Figure 7f). A unique 55 kDa positive signal was immunodetected in microglia-derived EV (lane 1) as well as in microglial cell (lane 2) protein extracts, confirming the origin of nGDF-positive EVs. No signal was observed on the same samples using secondary antibodies alone as negative control (lanes 3 and 4). Western blot analysis was performed on the same membrane using an anti-GAPDH antibody as a loading control (lanes 5 and 6). A double immunostaining was performed to establish a possible colocation of nGDF with Alix in nerve cord T24-h post lesion. Of interest, most of the EVs surrounding a neuron cell body resulted positive for nGDF (Figure 7g) as well as Alix (Figure 7h). The merge of both images demonstrates that the punctate signal immunopositive for nGDF is indeed related to EVs (yellow, Figure 7i). Some red structures are still visible suggesting that some EVs in ganglia would not contain nGDF.

**Figure 8:**
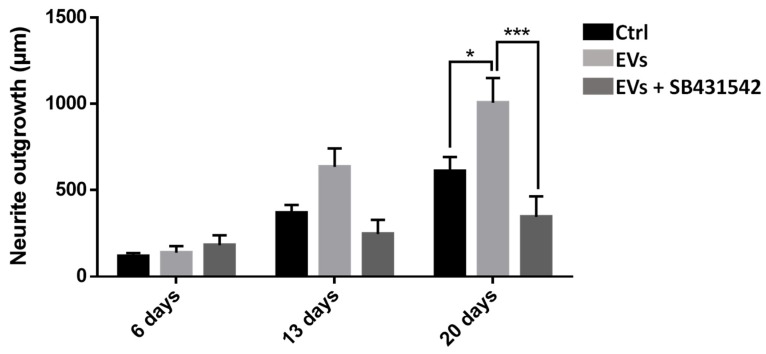
Influence of nGDF-dependent EVs in neurite outgrowth assays. Leech neurons were primarily cultured with either EV-enriched fraction, EV-enriched fraction + TGF-β signaling pathway inhibitor (SB431542) or control supernatants from enrichment procedure. After measures of neurite length, the EV-activated condition showed a significant higher outgrowth at day 20 compared to the control. The presence of SB431542 inhibitor in cells submitted to EV-activated condition showed a significant lower outgrowth at day 20 compared to EV-enriched fraction alone. Significance (* *p* < 0.05, *** *p* < 0.001) was calculated by ANOVA paired *t*-test (bar represents standard errors of mean).

To study the importance of nGDF in the EV-dependent dialog from microglia to neurons, neurite outgrowth assays using leech primary neurons compared the effects of microglia EVs under TGF-β signaling inhibition. Compared to control condition, the neurite outgrowth of leech neurons (day 6 to day 20) increased in the presence of microglia EVs (Figure 8). Of interest, the neurite outgrowth significantly decreased when neurons were submitted to a specific TGF-β type I receptor inhibitor although incubated with EVs. Thus, the results show that microglia EV-specific nGDF might be involved in microglia EV-mediated neurotrophic process.

## 3. Discussion

Extracellular vesicles (EVs) are gaining worldwide interest due to their presence in biological fluids, including urine [38,39], saliva [40], plasma [41,42], and cerebrospinal fluid [43,44]. They are produced by almost all cell types and organisms. Both exosomes and microvesicles (ectosomes) share common biologically active molecules, including proteins, lipids, and nucleic acids [22,23,24], and biogenesis mechanisms [25,45]. Therefore, this novel research field in cell-to-cell communication was investigated to better understand their ability to inform/affect recipient cells either in physiological or in pathological conditions. Addressing questions in basic research of this field may be quickly improved towards EV studies as biomarkers or therapeutic agents [27,28]. As mentioned before, in CNS, crosstalk between glia and neurons is crucial for brain integrity. Glia and neurons can communicate by releasing and receiving extracellular vesicles, which allows a synchronized regulation across long distances [46,47,48,49]. In this natural strategy, microglia play a crucial role [50].

In the present report, the results showed that the leech CNS produces and releases an important population of EVs, as revealed using Alix biomarker [34]. Alix is a cytosolic protein of the endosomal sorting complex. Its Bro1 domain binds with multivesicular body components (endosomal sorting complexes required for transport ESCRT-III proteins) which involves Alix in EV biogenesis [51]. The large production of EVs is at least exerted by microglia suggesting their important capacity to crosstalk with neighbor cells, including neurons. Indeed, the use of an intermediate filament, Gliarin, as a specific marker for glial cells [37] demonstrated that microglia-derived EVs strongly interact with neurons in vitro. The control experiments carried out in addition to anti-gliarin immunolabeling were specifically dedicated to highlight areas of the sample that can be revealed in a nonspecific manner. The single neuron culture (Figure 5b) and the secondary antibody alone as another control (Figure 5c) showed that the culture coating as well as the neuron cell body present a nonspecific signal. None of the two controls showed recognition of the extracellular vesicles interacting with the neurites, which demonstrates the specificity of these structures for the anti-gliarin antibody. An additional Western blot analysis using anti-gliarin definitely demonstrated that microglia-enriched EVs are actually positive for gliarin. Because gliarin is a glial marker in the leech [37] and because the experiments only used microglia, as glial population, in primary co-culture with neurons, all signals being immunopositive for gliarin are specific for microglial cells and the products that result therefrom. This glial marker was also used in this regard to exclude any observation of neuron-derived EVs, which would naturally occur in a paracrine manner. Thus, the present report showed a neuron-specific tropism of microglia EVs in vitro. Leech microglia are similar to vertebrate ones since they change their morphology for migrating towards lesions [14]. Under several chemotactic signals [14,15,16,17], microglia cells are the only cell type to reach the injury site then contributing to a specific axonal sprouting [13]. Therefore, elucidating interaction mechanisms between microglia and neurons is crucial to better understand neuroprotective events leading to nerve regeneration. In the tubular architecture of the leech nerve chain, the neuronal cell bodies are only located in ganglia while their axons are projected in connective tissues. Thus, a mechanical lesion in the middle of connectives allows the study of a specific crosstalk between accumulated microglia at the lesion site and damaged axons [7]. Other microglial cells, residing in ganglia, can dialog with neuronal cell bodies [8]. This strong interaction between microglia and neurons can be easily observed ex vivo and investigated in vitro by collecting and maintaining both cell types in primary cultures. In this context, the EV populations that are produced from activated microglia represent important molecular cargos supporting functional activities into recipient cells. The present report showed that leech microglia EVs might deliver neurotrophic messages to leech neurons. Even if EV-enriched fraction exhibits a higher effect than that of control supernatant on neurite outgrowth, we cannot assert that supernatant after the enrichment procedure are free of microglia-derived cytokines or trophic factors. These microglia-derived molecules might exert a positive effect in the control throughout the culture. However, we can confirm that such potential compounds from the supernatant do not artificially contaminate the EV-enriched pellet since this fraction was obtained after a supplementary washing and ultracentrifugation step. That is why, the EV fraction permitted to evaluate the natural neurotrophic effect of microglia through its EV release with no microglia-derived secretome. In physiological conditions, these microglia-derived products, including soluble factors and EVs, would synergistically exert a regulative effect on neurons. This communication is even more important since leech microglia EVs triggered a significant increase of rat PC12 cell differentiation. This EV-mediated dialog between leech and rat cells shows a possible recognition and use of common molecular mediators. This EV impact across species has already been observed between rat and human cells [52] and even between plant cells and murine intestinal macrophages [53]. Although the recognition and internalization processes need to be elucidated in those cross-species communications, both cases showed the regulation of specific target genes in recipient cells. It interestingly suggests that interactions between cells are more a matter of molecules than of species. Thus, it becomes possible to use the leech CNS as an alternative model to enrich EVs and characterize their contents. In this report, a member of TGF-β family has been identified in leech as a microglia EV content for in vitro regulation of neurite outgrowth. The results obtained by quantitative PCR show the presence of the *ngdf* transcript in freshly dissected leech microglial cells (T0h). Although the *ngdf* gene is constitutively expressed, the protein is not detectable at T0h either in microglial cells or in extracellular vesicles in ganglia ex vivo. The *ngdf* mRNA is still present in cultured (T24h) primary microglia. The increase of this transcript observed by q-PCR might be simply due to the mRNA accumulation or to a maturation state of cultured cells. It could also be linked to the stress induced by mechanical tissue dissociation and culture conditions. Interestingly, the presence of the *ngdf* transcript in the hours following cell dissociation is consistent with the appearance of a high amount of nGDF-positive EVs in ganglia cultured ex vivo for 6 and 24 h post-lesion. These data suggest that the in vivo production of nGDF by microglia and the release of their nGDF-positive EVs could be involved in neuron-microglia crosstalk upon stress conditions. The results also showed that Alix-positive EVs in ganglia would not always contain nGDF suggesting the presence of EVs with other functions and/or other cell origin. It is likely that vesicular populations may be modified as a result of time and cell activation conditions. Subsequent studies might specify the extent to which the vesicles are naturally modified even if the present study already measures their consequence on neurite outgrowth. Further studies will be dedicated to characterizing other proteins and RNA molecules in order to better understand the functions of microglia EVs in neuroprotective events following injury. Future studies would also specify whether nGDF-related molecules could be involved in the EV-dependent communication across species. Current studies in the laboratory have already identified protein and miRNA compounds from those leech EVs and investigate their influence in the dialog with neurons. It will help to identify a molecular cocktail involved in the neuroprotection. Those mechanisms, if shared with mammalian nerve cells, would bring a new insight into cell communications in healthy or pathological brains. Because EVs represent a powerful diagnostic and therapeutic approach, leech data would contribute to the experimental use of EVs as one strategy to detect or impact CNS pathologies.

## 4. Materials and Methods

### 4.1. Leech Central Nervous System Dissection

All protocols regarding the use of leeches were carried out in strict accordance with the French legislation and European Treaty, and in compliance with the Helsinki Declaration. The adult leeches *Hirudo medicinalis* were obtained from Biopharm (Hendy, UK). After anesthesia in 10% ethanol at 4 °C for 20 min, CNS were dissected out in a sterile Ringer solution (115 mM NaCl, 1.8 mM CaCl_2_, 4 mM KCl, 10 mM Tris maleate, pH 7.4) under a laminar flow hood. The CNS were then placed in 3 successive baths of Leibovitz L-15 medium (Invitrogen, Carlsbad, CA, USA), containing antibiotics (100 UI/mL penicillin, 100 µg/mL streptomycin, and 100 µg/mL gentamycin) for 15 min each and further incubated for 15 min in complete medium made of L-15 medium complemented with 2 mM L-glutamin, 100 UI/mL penicillin, 100 µg/mL streptomycin, 100 µg/mL gentamycin, 0.6% glucose, 10 mM Hepes.

### 4.2. Neuron and Microglial Cell Preparation

The whole CNS was placed in 35-mm Petri dishes with 200 µL of complete medium. Each ganglion was carefully decapsulated by removing the collagen layer enveloping the nerve cord with micro-scissors. The nerve cells, neurons (6–70 µm), and microglia (3–5 µm) were mechanically collected by gentle scraping and filtered through different size of filters for separating the population according to size. First, the dissociated tissue was filtered using a 100-µm mesh size strainer (PluriSelect^®^, Dominique Dutscher, Brumath, France) in order to eliminate tissue debris. Then, the eluate was filtered through 40, 20, and 6 µm strainers to separate large, medium, and small size neurons, respectively, and to collect microglial cells in the flow-through.

### 4.3. Cell Primary Culture

Nerve cells were centrifuged at 1200× *g* to eliminate cell debris remaining in the dissociation medium. The pellet was resuspended in complete medium supplemented with 10% exosome-depleted fetal bovine serum (FBS) Media (SBI System Bioscience, Palo Alto, CA, USA), and nerve cells were placed in a culture chamber (Lab-Tek chambered cover glass 4 well, Thermo Fisher Scientific, Waltham, MA, USA) coated with poly-d-lysine (Sigma-Aldrich, Saint Louis, MO, USA). They were maintained for at least 15 days in a humid environment at 15 °C, conditions routinely used for leech nerve cord or primary cell cultures. Half medium was carefully changed every 4 days.

### 4.4. Immunohistochemistry on Nerve Cord

After dissection, the nerve cord was cut into fragments of 4 ganglia. Each fragment was injured by crushing the two connectives between the 2nd and 3rd ganglia [7]. The nerve cord fragments were maintained for 24 h in culture in complete medium supplemented with 10% exosome-depleted FBS Media. The nerve cords were fixed with 4% paraformaldehyde at room temperature (RT) for 1 h. Tissues were then washed 3 times in PBS and permeabilized by a 24 h incubation at 4 °C in a 1% Triton ×100 solution diluted in phosphate buffered saline (PBS). To avoid nonspecific background staining, the nerve cord fragments were pre-incubated in blocking buffer (1% Triton, 3% Normal Donkey Serum (NDS) and 1% BSA/ovalbumin in PBS/glycine 0.1 M) for 8 h at 4 °C. They were then incubated overnight at 4 °C with specific primary antibody diluted in blocking buffer: either mouse polyclonal anti-human Alix antibody (dilution 1:500, ab88743, Abcam, Cambridge, UK) or rabbit polyclonal anti-human TGF-β1 antibody (dilution 1:100, ab92486, Abcam, Cambridge, UK). After 3 washes for 15 min in blocking buffer, the nerve cords were incubated 1 h at 37 °C in secondary antibody diluted in blocking buffer: donkey anti-rabbit immunoglobulin G (IgG) antibody conjugated to Alexa Fluor 488 (dilution 1:2000, Invitrogen, Carlsbad, CA, USA). They were then rinsed for 15 min three times in PBS and cell nuclei were counterstained by Hoechst 33342 fluorescent dye (dilution 1:10,000, Invitrogen, Carlsbad, CA, USA) for 20 min at 4 °C. Finally, nerve cords were mounted on slide with Dako Fluorescent Mounting Medium (Agilent Dako, Santa Clara, CA, USA). Control experiments were performed following the same immunostaining protocol without the primary antibody incubation.

### 4.5. Immunofluorescence on Nerve Cells

Primary culture at 15 days (neurons with or without microglial cells) were rinsed with PBS before fixation with 4% paraformaldehyde during 20 min and then washed 3 times in PBS. The poorly adherent microglial cells were lost during these multiple steps of washing. Nerve cells were incubated in blocking buffer (0.05% Triton, 1% Normal Donkey Serum (NDS) and 1% BSA/ovalbumin in PBS/glycine 0.1 M) for 30 min at 4 °C to avoid nonspecific background staining. Neurons were then incubated overnight at 4 °C with mouse monoclonal anti-gliarin antibody diluted in blocking buffer (dilution 1:500, kindly gifted by J. Johansen (Iowa State University, IA, USA)). After 3 washes for 10 min in blocking buffer, the nerve cells were incubated for 1 h at 37 °C with secondary donkey anti-mouse IgG antibody conjugated to Alexa Fluor 488 diluted in blocking buffer (dilution 1:2000, Invitrogen, Carlsbad, CA, USA). Neurons were rinsed with PBS and counterstained with phalloidin tetramethylrhodamine B for 30 min at 4 °C (5 µg/mL, sc 301530, Santa Cruz Biotechnology, Santa Cruz, CA, USA). Finally, after a last PBS washing, cells were mounted on a slide with Dako Fluorescent Mounting Medium (Agilent Dako, Santa Clara, CA, USA). Control experiments were performed following the same immunostaining protocol without the primary antibody incubation.

### 4.6. Image Acquisition

Slides, kept at 4 °C in the dark, were observed with a Zeiss LSM700 confocal microscope connected to a Zeiss Axiovert 200 M equipped with an EC Plan-Neofluar 40×/1.30 numerical aperture and an oil immersion objective (Carl Zeiss AG, Oberkochen, Germany). The image acquisition characteristics (pinhole aperture, laser intensity, scan speed) were the same throughout the experiments to ensure comparability of the results. Processing of the images was performed using Zen software and applied on the entire images as well as on controls.

### 4.7. Molecular Characterization

A *Hirudo medicinalis* draft genome was characterized in a Hirudinea Genomics Consortium as previously described [54]. Sequences were assembled from paired short reads using Velvet and PHRAP/CONSED algorithms [55,56] and managed with GlimmerHMM to get the predicted mRNA database [57]. The predicted mRNA sequences were submitted to a Local BLAST program using a human Alix amino acid sequence as reference [58]. The candidate sequence was then submitted to SwissProt databases using BLAST in order to specify similarities. From the putative mRNA sequence, specific primers were designed to get the natural and complete sequence by RACE-PCR from CNS total RNAs. Total RNAs were extracted from the CNS of 10 leeches. Dissected nerve cords were incubated in TRIzol^®^ reagent (Thermo Fisher Scientific, Waltham, MA, USA) and homogenized using tissue homogenizer (Precellys, Ozyme, Yvelines, France). The extraction of the total RNA was performed according to the manufacturer’s protocol and resuspended in RNase-free water. The extracted total RNAs were treated with RNA qualified (RQ1)-DNase1 (Promega, Madison, WI, USA) to prevent any contamination by genomic DNA. By using SMARTer^®^ RACE 5′/3′ Kit (Takara Bio, Mountain View, CA, USA), 2 µg of total RNA were used to construct 5′ and 3′ cDNA libraries according to the manufacturer’s instructions. Then nested PCR amplification reactions respecting Takara instructions were performed using a combination of forward and reverse primers framing Alix mRNA sequence (Fw 5′TAGATCGTGAATTGTTTTCAAATCATTC3′; Rw 5′TTATTTGTTAAGCCAAACCTTTAAATAATATAG3′). The reaction cycles were performed as follows: 94 °C for 1 min, followed by 40 cycles of 30 s at 94 °C, 30 s at 53 °C, and 2 min at 72 °C. The PCR products were ligated into the pGEM T-easy vector (Promega, Madison, WI, USA) and cloned into JM109 cells according to the manufacturer’s instructions. Finally, products were sequenced using BigDye Terminator v3.0 polymerization kit before detection on Genetic Analyzer (Applied Biosystems, Foster City, CA, USA). *Hm*Alix is now referenced as Genbank Accession Number MK060007.

### 4.8. Gene Expression Analysis

The microglia cells were collected, as described above, from the CNS of 10 leeches for each experimental condition and incubated in complete medium. Total RNAs were extracted with TRIzol^®^ reagent (Thermo Fisher Scientific, Waltham, MA, USA) according to the manufacturer’s protocol and resuspended in RNase-free water. Total RNAs were treated with RQ1-DNase1 (Promega, Madison, WI, USA) to prevent any contamination by genomic DNA. The cDNA library was generated from 2 µg of total RNA using random primers and Superscript III Reverse Transcriptase kit (Invitrogen, Carlsbad, CA, USA) in a final volume of 20 µL following the manufacturer protocol. The cDNAs were treated with RNase H (Promega, Madison, WI, USA) to optimize the amplification reaction product. Real-time quantitative PCR (q-PCR) were performed with the Platinum SYBR Green qPCR SuperMix (Invitrogen, Carlsbad, CA, USA) by combining 2 µL of cDNA template, 2 µL of primer mix (10 mM), and 25 µL of Platinum SYBR Green qPCR SuperMix-UDG in a final volume of 50 µL. Regarding leech *ngdf* gene, (Genbank Accession Number MH346328) specific primers were designed for the qPCR analyses (5′-TGCTTGTGGTTCTCGGACTC-3′ and 5′-TTTCGCTCTGATCTGCTGCA-3′). Specific primers were also designed for leech 18s ribosomal RNA (5′-GGAGGAGCGCGTTTATTAAG-3′ and 5′-GGGCACACACTTGAAACATC-3′) used as normalizer. The qPCR reactions were conducted on CFX 96 Real-Time System (Bio-Rad, Hercules, CA, USA) with the following conditions: 2 min at 50 °C (1 cycle), 2 min at 95 °C (1 cycle), 30 s at 95 °C, 30 s at 58 °C, and 30 s at 60 °C (39 cycles) followed by a final melting curve to control the amplified specificity. The expression level of the *ngdf* gene was compared between microglial cells 15 min (T0h) and 24 h post lesion (T24h). Experiments were done on triplicate samples in different sets of cDNA template. The analysis of *ngdf* relative gene expression was calculated using the 2^−ΔΔCt^ method [59]. Statistical analyses were performed by Paired *t*-test using GraphPad Prism 6.0 software. Statistical differences were considered to be significant if the *p*-value < 0.05.

### 4.9. Extracellular Vesicle Isolation

Centrifugations for extracellular vesicle isolation were all performed at 4 °C. Importantly, to limit degradation, isolated EVs were freshly processed without any frozen step. The content of each microglial cell culture well was transferred into 1.5 mL low-binding Eppendorf^®^ tubes (Hamburg, Germany) and centrifuged at 1200× *g* for 10 min to remove any cells. The supernatants (S1) were transferred to sterile tubes and centrifuged at 1200× *g* for 20 min to eliminate apoptotic bodies. This step was followed by filtration through a 0.22-µm filter to eliminate large vesicles and some apoptotic bodies possibly remaining in the supernatant. In order to pellet the EVs, the supernatants (S2) from all samples were pooled and transferred into a 10.4-mL polycarbonate bottle with cap assembly tubes (Beckman Coulter, Brea, CA, USA). The tubes were completed with PBS to a final volume of 9 mL and samples were ultra-centrifuged at 100,000× *g* for 90 min in a 70.1 Ti rotor, k-factor 36 (Beckman Coulter, Brea, CA, USA). Supernatants were removed (S3) and the EVs pellets were resuspended in 200 µL of PBS. The tubes were again filled with PBS to a final volume of 9 mL and samples were ultra-centrifuged at 100,000× *g* as previously described. Supernatants were removed and the EVs pellets (UC samples) were resuspended in 200 µL of PBS for further analyses. Nanoparticle tracking analysis was performed using a NanoSight NS300 instrument (Malvern Panalytical Ltd, Malvern, UK) according to the manufacturer’s instruction in order to visualize and count EVs.

### 4.10. Protein Extract

Microglial cell and EV protein extractions were performed with RIPA buffer (150 mM NaCl, 50 mM Tris, 5 mM EGTA, 2 mM EDTA, 100 mM NaF, 10 mm sodium pyrophosphate, 1% Nonidet P-40, 1 mM PMSF, 1× protease inhibitor) 30 min at 37 °C or at RT. Cell debris were removed by centrifugation at 1200× *g* for 20 min at RT. The supernatants were collected and the protein concentrations were measured using a Protein Assay (Bio-Rad, Hercules, CA, USA).

### 4.11. SDS-PAGE and Western Blotting

Protein extracts (30 µg) were homogenized (*v*/*v*) in 2× Laemmli sample buffer and loaded on a 12% polyacrylamide gel. Protein migration was conducted in TGS buffer (25 mM Tris pH 8.5, 192 mM Glycine, and 0.1% SDS) at 70 V for 15 min and 120 V for 45 min. The separated proteins were transferred on nitrocellulose membranes (AmershamTM HybondTM-ECL, GE Healthcare, Little Chalfont, UK). The membranes were incubated for 1 h at RT in blocking buffer (0.05% Tween 20 *w*/*v*, 5% milk powder *w*/*v* in 0.1 M PBS, pH 7.4) and then overnight at 4 °C with specific primary antibody diluted in blocking buffer: either mouse polyclonal anti-human Alix antibody (dilution 1:500, ab88743, Abcam, Cambridge, UK) or rabbit polyclonal anti-human TGF-β1 antibody (dilution 1:200, ab92486, Abcam, Cambridge, UK). A mouse monoclonal anti-rabbit GAPDH antibody (dilution 1:500, ab8245, Abcam, Cambridge, UK) was also used as loading control for secondary antibody membranes (see below). Membranes were washed three times in blocking buffer for 15 min, before incubation for 1 h at RT in secondary antibody diluted in blocking buffer (goat polyclonal anti-rabbit IgG antibody conjugated with horseradish peroxidase, dilution 1:20000, Jackson Immunoresearch, Cambridgeshire, UK). Finally, after two washes in PBS-0.05% Tween 20 and another one in PBS, immunolabelled proteins were revealed using the ECL Kit SuperSignal West Dura Chemiluminescent Substrate (Thermo Fisher Scientific, Waltham, MA, USA). Chemiluminescence acquisitions were performed with ImageQuant LAS-4000 mini system (Fujifilm, Tokyo, Japan). Negative controls were performed using secondary antibody alone. The control membranes with secondary antibody were then stripped. The membranes were rinsed three times with 0.05% PBS-Tween 20 for 5 min and incubated in stripping solution (0.2 M citric acid) for 30 min at RT. Then membranes were rinsed five times with 0.05% PBS-Tween 20 for 5 min to go to the incubation in the blocking buffer and were probed with a mouse monoclonal anti-rabbit GAPDH antibody (dilution 1:500, ab8245, Abcam, Cambridge, UK) as a loading control to confirm the properly loading of the protein extracts on the control experiments.

### 4.12. Transmission Electron Microscopy (TEM)

After microglial cell preparation (see above), cells were centrifuged at 1200× *g* for 10 min and included in 3.5% agarose (Low Melt agarose, Bio-Rad, Hercules, CA, USA). Cells were then impregnated overnight with 2.3 M saccharose in PBS at 4 °C to preserve cytoplasmic contents, and frozen under liquid nitrogen. Microglial cell blocks were cut in serial thin cryosections of 90 nm using a Leica AFS2 system (Leica Microsystems, Vienna, Austria) and collected on Formvar-carbon coated grids. Samples were finally included and contrasted for 15 min on ice with a 4% uranyl acetate and 2% methylcellulose mixture (1:9, *v*/*v*) and dried overnight. Concerning EV-enriched fractions from microglia cells (see enrichment method above), the samples were fixed in Karnovsky’s fixative (2% glutaraldehyde + 2% paraformaldehyde buffered with 0.1 M sodium cacodylate, 0.01% CaCl_2_, 0.01% MgCl_2_, 2% sucrose) and sample were deposited on Formvar-carbon coated grids by 3 adsorptions of 20 min in a wet environment. The grids were then stained with 4% uranyl acetate and 2% methylcellulose mixture (1:9, *v*/*v*) and dried overnight. Samples were observed on a JEOL JEM-2100 transmission electron microscope, respectively, at 75 kV or 200 kV (JEOL, Akishima, Tokyo, Japan). The acquisitions were made with GatanOrius SC200D camera. For immuno-electron microscopy, extracellular vesicles were purified by differential ultracentrifugation (see above) and the pellet frozen under liquid nitrogen. Sample sections and grids preparation were performed as described above. After 30 min of incubation in blocking solution (5% donkey serum, 0.2% BSA in PBS buffer), samples were exposed overnight at room temperature in mouse polyclonal anti-human Alix antibodies (1:100 in PBS/0.1%BSA, Ab88743). Control sections were incubated in the same buffer without primary antibodies. After washing in PBS, samples were incubated for 2 h at room temperature in appropriate secondary gold-conjugated anti-mouse IgG antibodies (particle size 10 nm; Jackson ImmunoResearch) diluted 1:100 in PBS. Following washing steps in a droplet of water, samples were stained in 0.5% uranyl acetate for 10 min. Electron microscopy observations were performed as indicated above.

### 4.13. Neurite Outgrowth Assays on Rat PC12 Cell Line

The rat PC12 adrenal pheochromocytoma cell line was purchased from the American Type Culture Collection (ATCC, Manassas, VA, USA). PC12 cells were cultured in a humidified atmosphere with 5% CO_2_ at 37 °C, in high glucose Dulbecco’s Modified Eagle’s Medium (DMEM) and supplemented with 2 mM l-glutamin, 10% heat-inactivated fetal horse serum (FHS), 5% heat-inactivated fetal bovine serum (FBS), 100 UI/mL penicillin, 100 µg/mL streptomycin (multiplication medium). Triplicate experiments were performed in 24-well plates, on cover glass coated with poly-d-lysine. PC12 cells were plated in multiplication medium (260,000 cells/well) for 24 h. Then the medium was changed for differentiation medium, where FHS concentration was reduced to 0.1% in DMEM supplemented with L-glutamin, penicillin and streptomycin, with or without (control experiments) presence of microglia EVs. At culture day 4, all the wells were completed with 100 µl of fresh differentiation medium. PC12 cells were then fixed at day 7 with 4% paraformaldehyde 20 min and counterstained with phalloidin tetramethylrhodamine B for 30 min at 4 °C to evaluate neurite length. Finally, after a last PBS washing, cells were mounted on a slide with Dako Fluorescent Mounting Medium and kept in the dark before acquisition. The neurite length was measured with Neurite Tracer ImageJ software program.

### 4.14. Neurite Outgrowth Assays on Leech Neurons

In order to perform statistically relevant experiments, the neurons were collected from 20 individual leeches at the same developmental stage and pooled in a homogeneous cell preparation to avoid individual polymorphism. From this cellular pool, the cells were distributed, homogeneously and in the same quantity, and were primarily cultured in individual plates in previously described conditions, in a complete exosome-depleted medium L15. After a 6-day culture, neurons were exposed to 10^6^ EVs/well, control S3 supernatant, or EVs + TGF-β signaling pathway inhibitor (20 µM SB431542, R&D Systems, Minneapolis, MN, USA) [60]. A complete medium renewal was done every 4 days. S3 permits to verify an eventual presence of neurotrophic factors in the microglia-conditioned medium. Image acquisition was realized with a microscopy station Nikon Eclipse Ti2 (Nikon, Minato, Tokyo, Japan). This station, equipped with a perfect focus system which automatically rectifies focus control for drift, was adapted for real time analysis. Acquisitions were realized at day 6, 13, and 20 of culture, each well was totally scanned, and a mosaic image was created for each condition. Only neurons presenting neurites at the 3 acquisition times were selected for neurite length measurement. The experiments performed in this study considered the neurite length of each neuron individually (*n* = 25) instead of only one global counting coming from all cells present in the culture chamber. Measures were independently performed using ImageJ software program on individual neurons so that every cell can be followed time after time. Measures were then analyzed with GraphPad software using a statistic test “two way ANOVA”, to compare different experimental conditions over time.
